# Targeting the Transcriptome Through Globally Acting Components

**DOI:** 10.3389/fgene.2021.749850

**Published:** 2021-09-16

**Authors:** Damien Parrello, Maria Vlasenok, Lincoln Kranz, Sergei Nechaev

**Affiliations:** ^1^ Department of Biomedical Sciences, University of North Dakota School of Medicine, Grand Forks, ND, United States; ^2^ Skolkovo Institute of Science and Technology, Moscow, Russia

**Keywords:** transcriptome regulation, epigenetics, transcription elongation, RNA pol II pausing, NELF model

## Abstract

Transcription is a step in gene expression that defines the identity of cells and its dysregulation is associated with diseases. With advancing technologies revealing molecular underpinnings of the cell with ever-higher precision, our ability to view the transcriptomes may have surpassed our knowledge of the principles behind their organization. The human RNA polymerase II (Pol II) machinery comprises thousands of components that, in conjunction with epigenetic and other mechanisms, drive specialized programs of development, differentiation, and responses to the environment. Parts of these programs are repurposed in oncogenic transformation. Targeting of cancers is commonly done by inhibiting general or broadly acting components of the cellular machinery. The critical unanswered question is how globally acting or general factors exert cell type specific effects on transcription. One solution, which is discussed here, may be among the events that take place at genes during early Pol II transcription elongation. This essay turns the spotlight on the well-known phenomenon of promoter-proximal Pol II pausing as a step that separates signals that establish pausing genome-wide from those that release the paused Pol II into the gene. Concepts generated in this rapidly developing field will enhance our understanding of basic principles behind transcriptome organization and hopefully translate into better therapies at the bedside.

## Introduction

Treatments of cancers should ideally be tailored to their specific molecular signatures. The central place and complexity of transcription regulation in normal and cancer cells offer tantalizing opportunities for precise targeting (Bushweller, 2019; Van Hoeck et al., 2019; Malone et al., 2020). Many anticancer drugs in use and in development today target the transcriptional machinery or epigenetic regulators (Villicaña et al., 2014; Mohammad et al., 2019; Park and Han, 2019; Laham-Karam et al., 2020). However, rather than specific transcription factors, many drugs work against factors with broad-spectrum functionality including epigenome modifiers and components of the basal transcriptional machinery (Bywater et al., 2013). This targeting strategy draws from long-standing observations that perturbation of general or globally acting factors often results in distinct cell type specific effects for reasons that remain poorly understood (Ptashne, 2013). Because many targets of anti-cancer therapies have broad or essential roles in normal cells, their use remains heavily based on empirical findings. Targeting specific factors such as transcription factors causative of certain cancers is becoming feasible (Sievers et al., 2018; Duffy and Crown, 2021), but is limited by an uncertainty in how these and other factors may function in different cellular contexts.

Transcription is the first step in expression of genes and genomes. The combined activity of some 20,000 human genes results in genome-wide RNA transcriptome patterns that reflect the biology and define the identity of every cell. Despite the flood of technologies describing the transcriptomes with increasing precision, our understanding of gene regulation remains fundamentally based on the knowledge gained from studies of individual genes. A long-standing gene-centric paradigm describes regulation by sequence-specific transcription factors that serve as repressors and activators, in contrast to factors broadly involved in the process of transcription that are considered basal or general (Nikolov and Burley, 1997; Juven-Gershon and Kadonaga, 2010). This paradigm does not explain network-level events especially the molecular rules governing interactions among thousands of genes in different cell types.

Two features of Pol II transcription are of note. On the one hand, the Pol II machinery is constantly modulated by a host of activating and repressing inputs that connect transcription to the environment within and outside the cell. These second to minute-scale events underlie rapid responses to stimuli and have generated the bulk of our understanding of transcription regulation in cellular responses to environmental triggers such as heat shock, hormones such as estrogen, or innate immune responses (Adelman et al., 2009; Hah et al., 2011; Mahat et al., 2016). In addition, metazoan cells have a special ability to form distinct stable steady states and undergo regulated transitions between them. These transitions take place on longer timescales, lasting hours to years, and involve predefined programs that are commonly visualized through the concept of the epigenetic landscape (Waddington, 1957). These transitions underlie development and differentiation of normal cells and involve transcriptional and epigenetic control mechanisms and factors that can be ectopically activated in cancers (Rousseaux et al., 2013).

Targeting strategies should benefit from better understanding of principles that govern the transcriptomes. This problem can be conceptually narrowed down to defining the molecular interactions that link individual genes within transcriptional networks. Given the overall conservation of the RNA polymerase II machinery (Hampsey, 1998), mechanisms that drive this quantum leap in complexity in higher organisms presumably do not involve too many additional players and instead must rely on repurposing of existing components. In this essay, we discuss some of the challenges in targeting the transcriptional machinery and suggest a potential avenue for improving the precision of broad-stroke interventions.

## Pervasive Uncertainty in Targeting Cellular Components

In this section we describe some of the challenges in targeting the transcriptional machinery. These arise not only from unintended effects of drugs, which can be improved by identifying better targets and better drug design, but also from the inherent uncertainty of transcription regulation in different cellular contexts.

### Targeting the Transcription Machinery: Knocking on the Black Box

Transcription is the ultimate target of numerous anticancer drugs that act on Pol II or the epigenetic machinery. Cancer targeting aims to either kill or reprogram cells into more benign states (Gong et al., 2019). To gain selectivity over normal cells, targeting strategies exploit distinct properties of cancer cells. One property is addiction to transcription (Bradner et al., 2017), which increases the demands of cancer cells for Pol II activity and makes them more sensitive to its inhibition. Inhibitors of general transcription factors such as TFIIH and P-TEFb have been used (Villicaña et al., 2014; Wang et al., 2015; Sava et al., 2020). Known oncogene transcription factors such as c-Myc, KRAS, etc, are tempting targets because of their key roles in cancer initiation and progression (Hallin et al., 2020; Madden et al., 2021). However, some of these factors have been considered undruggable or difficult to target for various reasons including their critical roles in normal cells and/or difficulty to specifically target interactors as compared to enzymes (Lazo and Sharlow, 2016; Zhang et al., 2018). Recent studies suggest that these challenges will be at least to some extent overcome (Duffy and Crown, 2021; Trkulja et al., 2021; Wang et al., 2021). However, the highly changeable nature of cancers that can outselect therapies will always remain a formidable caveat.

A second property of cancer cells is broad dysregulation of the transcriptional machinery (Bywater et al., 2013; Lee and Young, 2013), which alters the requirements of cancer cells for its components and leads to unusual sensitivity to inhibition of certain factors. Several classes of epigenetic drugs are in development or already on the market, with the more common including Histone Deacetylase (HDAC) Inhibitors, Histone Acetyltransferase (HAT) inhibitors, Bromodomain Inhibitors, DNA methylation inhibitors, etc. These and others are described in detail in reviews elsewhere, for example, in (Heerboth et al., 2014; Mohammad et al., 2019; Nepali and Liou, 2021). Broad-stroke targeting has been rather successful in generating drugs, placing a burden on better understanding of when to target distinct components.

A third property of cancer cells is altered expression of genes outside of those involved in transcription. Differentially expressed genes are often marked as sources for therapeutic targets. Identifying therapeutic targets is perhaps the most common justification for basic studies over the years. Increasing our understanding of how different types of cancers work has indeed resulted in identification of targets including surface and nuclear receptors, kinases, etc (Kannaiyan and Mahadevan, 2018; Zhao et al., 2019; Skidmore et al., 2020). While revealing molecular mechanisms of various processes and perhaps holding the keys to successful therapies in a long run, translation of these findings into therapies takes years with no guaranteed success. The ability to tailor a drug to a living cancer patient therefore remains limited (Krzyszczyk et al., 2018).

### Ambiguous Roles of Transcription Factors

Identifying causative factors for precise targeting of cancers is an attractive goal that has met serious challenges (Vishnoi et al., 2020). Cancers with well-known etiology such as fusion protein driven pediatric cancers remain difficult to target (Uren and Toretsky, 2005; Brien et al., 2019). This uncertainty is only amplified in adult cancers (Dawson et al., 2011; Fung et al., 2015; Shan et al., 2017; Winters and Bernt, 2017). One major reason behind this uncertainty is that the functions of individual factors can be dramatically altered across cell types and different individuals. This property may be inherent to transcription factors themselves and might not always be controllable.

DNA-binding transcription factors are commonly labeled as either activators or repressors (Wolffe et al., 1997). It is becoming increasingly clear, however, that most if not all transcription factors can, and likely do, function both as repressors and activators. There are several possible reasons for this duality. First, a factor itself may play different roles at the same loci. A number of transcription factors involved in *Drosophila* development and stimulus responses in human cells show default repression of target genes unless activated, usually by a co-factor, thereby appearing both as repressors and activators (Barolo and Posakony, 2002). In *E. coli* bacteriophage T4, the transcription factor gp33 causes default repression of late promoters, but becomes their potent co-activator in the presence of its specific co-factors (Kolesky et al., 2002; Nechaev and Geiduschek, 2006). *Drosophila* Hunchback and Dorsal (Pan and Courey, 1992; Dubnicoff et al., 1997; Bauer et al., 2010) serve as repressors or activators depending on co-factors on different promoters to drive highly coordinated embryo development (Staller et al., 2015). These well studied examples show that switching between a repressor and an activator in principle does not require complex changes.

Dual functions of transcription factors may arise through other mechanisms. One involves distinct activities for different isoforms of the same gene. Given a large number of known gene isoforms and frequent creation of new gene isoforms in cancers (Belluti et al., 2020), alternative splicing may be a significant contributor to the ambiguity of transcription factor designation, at least at the level of a gene (Walker et al., 1996). The same factors may also have different roles because they function in distinct complexes. The Polycomb Repressive Complex 2, PRC2, introduces the H3K27 histone mark with essential roles in development, cell differentiation, and cancer (Aranda et al., 2015; Schuettengruber et al., 2017; Healy et al., 2019). Ezh2 is the catalytic component of PRC2 responsible for introducing the mark. Ezh2 has been also shown to activate transcription in a separate role that does not involve its catalytic activity and is independent of PRC2 complex (Kim et al., 2018). Ezh2 role as an activator involves the binding at a promoter of a target (AR) gene as a DNA-binding transcription factor.

While the *Drosophila* factors have been well known to have dual roles, as more studies become available in human systems, even long-studied factors “acquire” opposing functions (Ip, 1995). For example, Snail is a conserved member of a family of E-box motif binding transcription factors that is involved in development though its role in the epithelial-mesenchymal transition with close relevance to cancer metastasis (Alberga et al., 1991; Carver et al., 2001; Peinado et al., 2004). Snail was initially considered to be exclusively a transcriptional repressor that binds to target gene promoters such as E-Cadherin. However, Snail was later shown to also activate genes in *Drosophila* during mesoderm development (Wu et al., 2017). Conversion of Snail from a repressor to an activator was shown to involve acetylation of Snail by the CREB-binding protein (CBP) (Hsu et al., 2014). Post-translational modifications can convert transcriptional repressors to activators (Mosley et al., 2003; Zhang et al., 2012), suggesting that this mechanism may be used in cancers as well. The duality of transcription factor roles as activators and repressors, therefore, is likely to be their inherent property rather than an exception.

Another major mechanism that can contribute to dual roles of transcription factors has to do not with their direct function, but with compensatory changes in the rest of the cell. On a short time scale, such as during rapid responses to stimuli, these changes may be driven by redistribution of cellular machinery components (Bregman et al., 1995). Such effects are considered secondary or nonspecific and are not well understood, but are pervasive and may be just as important as direct roles of transcription factors. For example, it is common for experimental perturbation of a factor by assays such as RNA-interference to cause both activation and repression of gene cohorts regardless of its actual mechanism of action. One possible exception is the transcriptional amplifier c-Myc that supports unbiased amplification of gene activity from all promoters genome-wide (Lin et al., 2012; Nie et al., 2012). Which genes are indirectly activated or repressed through secondary interactions should depend on the cellular context of individual cancers. The apparent dual roles of P53 tumor suppressor may fall into a similar category. The transcription factor p53 is widely considered to be an activator. Several studies, however, have proposed p53 as a direct repressor of genes (Banerjee et al., 2009; Allen et al., 2014). Its repressor role is controversial and has been suggested to be indirect (Fischer et al., 2014).

The ambiguity of functional designations for transcription factors extends to their phenotypic classification as oncogenes versus tumor suppressors (Shen et al., 2018; Datta et al., 2020), which may or may not be connected to their molecular mechanisms of action. The duality of transcriptional effects as well as cancer targeting outcomes persists through all levels of transcription factor function. These opposing functions are inherent to transcription factors and might not be separable even by specific targeting.

### Epigenetic Marks – A Knot Around Transcription

Epigenetic marks are known to be frequently altered in cancers (Jones and Baylin, 2007; Baylin and Jones, 2011; Bennett and Licht, 2018), making potentially reversible epigenetic reprogramming an attractive targeting strategy (Jin and Kim, 2017). There are caveats, however. First, known epigenetic marks have a broad scope, either covering large regions of the genome or distributed across multiple punctate regions, limiting the specificity of direct targeting. Second, many known histone marks are closely tied to transcription, either associated with repressed or active states of the nearby genes or regulatory elements such as enhancers (Henikoff and Shilatifard, 2011; Li et al., 2012; Kang et al., 2020) through mechanisms that remain to be fully understood. For example, histone H3 Lysine 4 trimethylation (H3K4Me3) preferentially marks active promoters, whereas monomethylation (H3K4Me1) mark appears to prefer regions outside of promoters including active and poised enhancers (Heintzman et al., 2007), and might have to do with transcriptional memory (Saeed et al., 2014; Bae and Lesch, 2020). Even with these well studied marks there are overlaps between distinct elements such as promoters and enhancers and the rules behind their deposition merit further studies (Pekowska et al., 2011; Scruggs et al., 2015; Soares et al., 2017). Histone H3K27 acetylation is commonly used to profile open genomic regions including active enhancers (Creyghton et al., 2010), but its functional roles remain not fully clear (Zhang et al., 2020). Acetylation patterns of various histones may be a good predictor for various types of regulatory elements (Rajagopal et al., 2014). Because genes and other regulatory regions are hotspots for multiple epigenetic marks with at least partial redundancy (Takeshima et al., 2009; Benveniste et al., 2014; Ahsendorf et al., 2017), targeting individual marks inevitably affects other marks and possibly the entire transcriptome.

The histone code hypothesis (Strahl and Allis, 2000) implies that the patterns of covalent histone modifications on a gene should reflect its dynamic regulatory state, likely contributing to widespread interest in epigenetics. Indeed, some histone modifications can be uncoupled from transcription activity. The conserved Polycomb Group (PcG) and Tritorax group of genes (trxG) complexes play crucial roles in development and introduce, respectively, repressive and activating histone modifications. The so-called bivalent genes that simultaneously harbor repressive and activating marks are poised for fate commitment in development and differentiation (Bernstein et al., 2006; Vastenhouw and Schier, 2012). The histone H3K36 modifications in the gene body regions may regulate alternative splicing (Kim et al., 2011). The histone H3K9 methylation, a repressive mark associated with heterochromatin, may be involved in arranging chromatin domains at the nuclear periphery (Towbin et al., 2012; Bian et al., 2020), protection against mechanical damage of the nucleus (Nava et al., 2020), remodeling of chromatin domains during differentiation (Wang et al., 2018; Burton et al., 2020) and maintenance of cell identity (Nicetto and Zaret, 2019). Many known histone modifications do not have clearly assigned functions yet, and will likely generate new findings (Tan et al., 2011).

**FIGURE 1:**
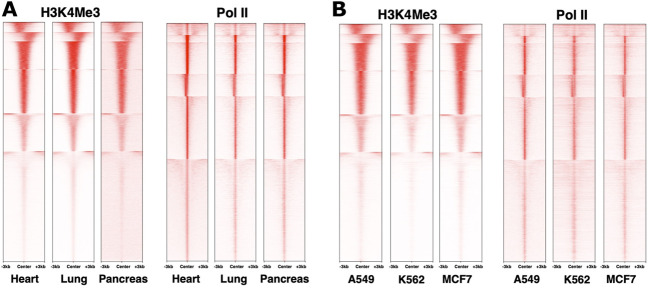
Similarity in genome-wide distributions of Pol II and histone marks across distinct systems. ChIP-sequencing datasets from the ENCODE database (Davis et al., 2018) for human heart, lung and pancreas normal tissues **(A)** and A549, MCF7 and K562 cancer cell lines **(B)** were used to plot density heatmaps around peak regions ± 3 kb from a peak center using *computeMatrix reference-point* (version 3.3.0) and *plotHeatmap* (version 3.3.0) with k-means clustering (k = 6). The numbers of peaks are 22,944 for Pol II and 28,287 for H3K4Me3. The datasets used were ENCSR901SIL, ENCSR701FGA, ENCSR876DCP, ENCSR336YRS, ENCSR033NHF, ENCSR610EFT, ENCSR000DMZ, ENCSR388QZF, ENCSR000DMT, ENCSR203XPU, ENCSR668LDD, ENCSR985MIB, respectively.

When considering the dynamics of chromatin modifications, it is important to distinguish differences across loci within a cell type from differences at the same locus across cell types. Different genes in the same cell type clearly show distinct epigenomic patterns (Ernst and Kellis, 2010). However, across cell types, genome-wide patterns for histone modifications that we examined (ENCODE et al., 2020), at least in bulk experiments, appear to be similar for the same loci (Figure 1 and not shown). This is mirrored in Pol II distribution as well (Figure 1), consistent with an earlier study, for example, (Day et al., 2016). Even examining a study that highlighted differences between epigenomes, most of the epigenomic features on a given gene are quantitatively similar (Yen and Kellis, 2015). Differences in epigenome patters between cell types may therefore be in relatively subtle shifts in balance among different marks (Gopi and Kidder, 2021), for example, in the breadth of regions harboring the marks (Benayoun et al., 2014), possibly representing distinct overall states of the nucleus or its compartments (Zhao et al., 2007), or distinct signaling pathways (Yen and Kellis, 2015). Comparing epigenetic marks remains difficult at this level due to quantitative limitations of omics technologies. Future studies employing emerging technologies and better integration of datasets should reveal new insights into the tightly interconnected workings of epigenetic marks. For example, recent work using a mouse model identified epigenetic reprogramming as an essential step for the initiation of pancreatic cancer, wherein cells carrying certain oncogenic mutations require an environmental insult that causes epigenetic changes and triggers cancer cell fate (Alonso-Curbelo et al., 2021). To the best of our knowledge, this study is among the first to directly demonstrate a role for an epigenetic “hit” to trigger carcinogenesis. Finding the reasons behind why some cells are more sensitive to epigenetic reprogramming by drugs or environment, and identifying the weak points for their reprogramming, is an exciting direction to explore.

### Multiple Sides of the Cellular Context

Cancers readily repurpose mechanisms that normally govern cell state transitions in differentiation, development, and responses to stimuli. Targeting of cancers borrows some of the overall concepts from the stem cells. Despite major advances in the stem cell field, reprogramming of stem cells rarely if ever approaches one hundred percent and can generate heterogeneous populations (Paik et al., 2018; Terryn et al., 2018). This heterogeneity, which is expected to be even higher in cancer cells, is likely to affect reprogramming and/or response to drugs. A recent study compared the effect of c-Myc on distinct fates of murine pre-B cells: transdifferentiating into macrophages and their reprogramming into iPSCs (Francesconi et al., 2019). Cells with high Myc activity reprogrammed to iPSCs more efficiently than transdifferentiated into macrophages, whereas cells with low Myc, in contrast, transdifferentiated readily, but failed to reprogram. That the levels of one factor can dramatically influence cell fate decisions highlights the importance and complexity of the cellular context.

Despite sharing certain features such as immortality and tendency for dedifferentiation, even recently transformed cells, without high if any mutation load, can readily deviate from normal cellular programs. A recent study showed that overexpression of the RAS oncogene in wild type mouse embryonic fibroblasts (MEFs) enhanced their dedifferentiation, but transformation of the same cells by deleting p53 or Arf tumor suppressors precluded it (Ferreirós et al., 2019). Similar interactions and context dependency were observed for histone marks as well (Nagaraja et al., 2019; Vidal et al., 2020). These data indicate that cancer cells diverge into distinct epigenetic programs, and likely change their cellular context, at the early stages of transformation. Thus, relatively small changes can lead to different responses to the same signal. These and other studies highlight the uncertainty that the cellular context can affect the apparent mechanism of transcription factor action as well as functional outcomes of its targeting.

## A Common Framework of Transcriptome Organization

In this section we discuss a genome-wide view of transcription noting overall similarities in gene expression and highlighting potential mechanisms that might be pertinent for understanding transcriptome regulation.

### The Transcriptome Rests on DNA

mRNA profiling has been widely used to identify genes with differential expression. Genome-wide gene expression patterns have been proposed as a basis to classify cancers (Perou et al., 2000). RNA-sequencing is arguably the easiest omics tool from the user’s point of view today, and some thousands of mRNA datasets are publicly available. Comparing RNA profiles of publicly available datasets of human tissues, a majority of transcripts are classified as present in all or in most tissues (Uhlén et al., 2016), indicating that, at least in bulk experiments, most genes show similar patterns of expression across distinct cell types. Despite substantial differences, cancer cells and primary tumors show unexpectedly high correlation of RNA signal even among distant cancer types (for example, (Yu et al., 2019)). Comparison of RNA profiles showed that the group of genes that are differentially expressed is rather similar between distant cell lines (Crow et al., 2019). Analysis of gene expression across several species showed that variation in expression among different genes exceeds that of the same gene in different conditions (Zrimec et al., 2020). Accordingly, genes that account for differential transcriptome profiles are relatively few and seem to fall into categories related to stress responses and immune function regardless of the cell type (Crow et al., 2019). A small number of genes may be sufficient to classify cancer subtypes as, for example, is done for breast cancers (Parker et al., 2009). RNA-sequencing in cancers is just as, if not more frequently used for the detection of exonic mutations (PCAWG et al., 2020). These observations point to overall similarity of gene expression states across the genome, that is, imply a fundamentally common transcriptome structure in human cells.

One way to breach the boundaries of transcription control is through changing gene copy numbers. Local and chromosome-wide changes of DNA copy number is a frequent occurrence and is one of the hallmarks of cancer (Hanahan and Weinberg, 2011). Changes in copy number enable cancer cells to alter expression of genes without any other regulatory inputs (Shao et al., 2019). This may reflect a fundamental property of mammalian transcriptomes wherein changes in gene copy numbers are not by default compensated (Disteche, 2016) and can alter the entire transcriptome. Indeed, genetic haploinsufficiency is associated with many diseases (Han et al., 2018). Dosage compensation is best known in X-chromosome inactivation (Brockdorff and Turner, 2015). Forced reactivation of the inactive X-chromosome copy by knocking down Xist levels in mice results in an increase of total X-linked gene expression (Yang et al., 2016) that can lead to cancer (Yildirim et al., 2013). Interestingly, autosomal polysomy in human cells can be compensated at the level of protein, but not at the level of RNA (Stingele et al., 2012). Sensitivity to DNA copy number raises an intriguing possibility that the transcriptome may be fundamentally structured by the process of transcription rather than its products.

### Pol II Pausing – A Common Step in Complex Organisms

**FIGURE 2:**
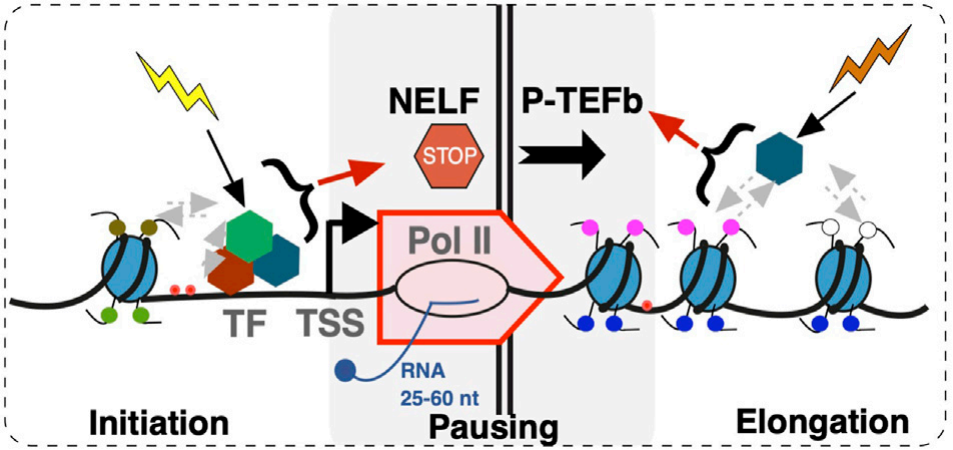
A scheme of events during early transcription elongation. Early transcription elongation within 25–60 nucleotides (nt) from the Transcription Start Site (TSS) is a step integrating upstream and downstream regulatory inputs. Upstream inputs from multiple factors including transcription factors (TF) and epigenetic marks result in transcription initiation at the promoter and culminate in NELF-dependent pausing. DSIF complex is not included in the model. Pause release into transcription elongation is dependent on P-TEFb. Uncoupling of transcription initiation and elongation at the site of pausing through mechanisms such as promoter proximal termination contributes to transcriptome organization.

Widespread accumulation of Pol II signal at promoter regions of genes has been well documented (Kim et al., 2005). Rather than preinitiation complexes, this signal comes largely from elongating Pol II that began RNA synthesis, but paused within the first ∼50 nucleotides (Pugh and Venters, 2016) (Figure 2). Because of its proximity to gene transcription start sites, the prevalence of Pol II pausing across the genome became clear only as technologies attained sufficient resolution (Muse et al., 2007; Zeitlinger et al., 2007; Nechaev et al., 2010). Perhaps because Pol II pausing was originally described on inducible genes such as MYC and heat shock HSP70 (O'Brien and Lis, 1991; Spencer and Groudine, 1990; Krumm et al., 1995), it was long believed to be a specialized mechanism that prepares highly inducible genes for activation. However, based on analyses of short RNA transcripts, Pol II pausing signatures are not confined to inducible genes, but are present on all genes, and likely accompany all transcription, whether initiating at or outside of promoters, including divergent transcription, intergenic transcription, enhancers, etc (Scheidegger et al., 2019). This makes pausing unlikely to be a mechanism that universally prepares genes for activation, although it likely contributes to it (see below) (Muse et al., 2007). Many details of Pol II pausing such as its relationship with the burst mode of transcription (Corrigan and Chubb, 2014; Fukaya et al., 2016; Tunnacliffe and Chubb, 2020) and its dynamics remain to be established. Whether Pol II pausing can be bypassed for individual transcription events or not, at least a certain percentage of Pol II complexes appear to undergo pausing at every start site of transcription (Scheidegger et al., 2019). The so-called “nonpaused” genes still show the same small RNA pausing signatures in terms of their size distributions, but are either less active or have a lower pausing index (Muse et al., 2007; Zeitlinger et al., 2007) (or higher traveling ratio (Rahl et al., 2010)) compared to other genes (Nechaev et al., 2010; Scheidegger et al., 2019). Examination of genome-wide datasets shows that different genes within a dataset show higher or lower pausing index, but these signatures, just like some histone marks, are overall stable across cell lines (Figure 1). These observations reinforce a notion on fundamental conservation of the human transcriptome and also raise a question about roles of pausing in regulation.

In considering potential biological roles of promoter-proximal Pol II pausing, it might be of significance that Pol II activity at this site is controlled by at least two distinct groups of factors: those that establish pausing and, on the other hand, those that release the Pol II from the paused state (Figure 2). The Negative Elongation Factor (NELF) is a five-subunit complex (Yamaguchi et al., 1999) that in conjunction with 5,6-dichloro-1-β-d-ribofuranosylbenzimidazole sensitivity-inducing factor (DSIF) (Yamaguchi et al., 2001) is sufficient to cause Pol II pausing *in vitro* (Renner et al., 2001; Wu et al., 2003). NELF is absent from yeast and *C. elegans*, indicating that NELF-dependent pausing is a function of higher organisms. Budding yeast *S. cerevisiae* do show Pol II accumulation at promoter regions at least at some genes (Radonjic et al., 2005). High-resolution nascent RNA analysis shows major differences between budding and fission (*S. pombe*) yeast, with the latter showing pausing signatures at a significant proportion of genes (Booth et al., 2016). However, fission yeast show clear differences from *Drosophila* and mammals in terms of the +1 nucleosome positioning presumably due to absence of NELF. These observations indicate that Pol II pausing is more prevalent and may be more tightly regulated in higher organisms.

Targeting of individual NELF subunit alters the levels of its other subunits (Sun et al., 2008), indicating that NELF components function as a complex. NELF is downregulated in breast cancers (Sun et al., 2008), but is potentially oncogenic in other cancers including prostate (Yun et al., 2018), liver (Dang et al., 2017; El Zeneini et al., 2017) and pancreas (Han et al., 2019). These observations indicate that the functional outcome of NELF perturbation is defined by the cellular context. The Positive Transcription Elongation factor B (P-TEFb) (Marshall and Price, 1995) is a two-component complex consisting of a cyclin T1, which can be substituted with cyclin T2 or possibly cyclin K, and a cyclin-dependent kinase 9 (CDK9) (Peng et al., 1998; Peterlin and Price, 2006; Kohoutek, 2009). P-TEFb releases the paused complex into productive elongation by phosphorylation of several proteins including DSIF subunit Spt5 at several sites (Wada et al., 1998; Kim and Sharp, 2001; Yamada et al., 2006; Parua et al., 2020), NELF (Fujinaga et al., 2004; Lu et al., 2016) and Pol II C-terminal domain at Ser-2 residues (Marshall et al., 1996). P-TEFb appears to be more conserved than NELF. Yeast may have more than one kinase (Hsin and Manley, 2012), although mammalian homologs of these kinases such as CDK12 appear to phosphorylate Pol II Ser-2 during elongation downstream of pause release by P-TEFb (Bartkowiak et al., 2010; Tellier et al., 2020). Neither NELF nor P-TEFb are essential for Pol II enzymatic function *in vitro*, but do appear to be essential for proper transcription *in vivo* (Sun et al., 2011; Aoi et al., 2020; Fujinaga, 2020). P-TEFb is a hub for regulatory inputs for multiple factors including c-MYC and NF-kB, and possibly many others (Zhou et al., 2012; Mahat et al., 2016; Aoi et al., 2020). The requirement for distinct essential factors individually controlling the on and off rates of a Pol II complex that is already committed to elongation is notable.

## Regulation by Globally Acting Factors

Here we discuss how transcription can be regulated by essential factors at the site of promoter-proximal Pol II pausing.

### Encoding the Change in the Static Genome

One genome must specify the entire structure and dynamics of the transcriptomes for in every cell, but the rules of how it does so remain obscure. It is evident, however, that promoters, including core promoter basal sequence elements such as the TATA box, initiator motif, Downstream Promoter Element, etc, do not merely serve as a passive platform for the binding of regulatory factors, but actively shape the outcomes of regulatory inputs (Butler and Kadonaga, 2002). Flexibility of promoters is fundamental and starts with bacteria: in *E. coli*, no single promoter, including for the highest expressed rRNA genes, contains a full consensus sequence of basal elements (Bervoets and Charlier, 2019). Instead, promoters are pre-wired to require additional inputs for highest-level transcription and thus to be inherently controllable. In eukaryotes, properties of promoters depend on the presence of distinct basal sequence elements (Butler and Kadonaga, 2002). Mechanisms for such selectivity may include preference for distinct cohorts of general transcription factors, localization within the nucleus, or interaction with enhancers (Juven-Gershon and Kadonaga, 2010; Zabidi et al., 2015; Russo et al., 2018). Differences in core promoter properties should lead to differences in their responses to the same signal, thereby creating patterns that can be complex especially with multiple signals. In this regard, about ∼350 human genes were recently noted to contain 5′-untranslated regions (UTRs) that show conservation across vertebrates, especially among specific categories of homeobox genes, kinases and genes involved in neurogenesis (Zuccotti et al., 2020).

Apart from differences between promoters, another question is whether and how the genome enables the same promoters to assume distinct regulatory states. In *Drosophila*, transcription start sites of regulatory genes contain sequences that favor both Pol II pausing and nucleosome binding at their transcription start sites (Gilchrist et al., 2010), which result in, respectively, active and repressed gene states. Housekeeping gene promoters, in contrast, do not contain these marks. By favoring mutually exclusive marks leading to distinct regulatory states, some promoters are intrinsically primed to be regulated. How these dynamic states are encoded in human promoters, which are highly enriched in CpG sequences, remains to be determined.

### Transcriptional Responses Expose the Transcriptome

Global requirement for NELF- and P-TEFb raises a question about their functional relationship at the site of Pol II pausing. It has been proposed that activation of genes should proceed through pause “release,” that is, relatively increased P-TEFb activity (Boehm et al., 2003; Hah et al., 2011; Liu et al., 2015; Mahat et al., 2016). Sustained transcription activation must also recruit additional Pol II, leaving a question as to how Pol II recruitment and pause release are related. A consistently rigid connection between pausing establishment and release would not be conducive for regulation. Evidence suggests that promoter Pol II recruitment and pause release can indeed be uncoupled. Chemical inhibition of P-TEFb - dependent Pol II pause release by flavopiridol causes accumulation of Pol II at promoters of active genes (Rahl et al., 2010; Henriques et al., 2013; Jonkers et al., 2014). Work by the Lis lab on heat shock response demonstrated that genes repressed by heat shock can accumulate Pol II signal at promoters (Mahat et al., 2016). Global Pol II accumulation at promoters has also been shown during acute oxidative stress response (Nilson et al., 2017). By demonstrating that Pol II pausing takes place even when P-TEFb activity is perturbed, these studies show that pausing establishment and release can be functionally uncoupled, and that this connection can be regulatory. Conversely, changes in Pol II recruitment without affecting pause release are possible as well. First, gene activation can take place through increased Pol II recruitment to promoters without changes in pause release (Samarakkody et al., 2015). Second, the Shilatifard lab showed that pause release can take place without additional Pol II recruitment when Pol II is being cleared from promoters prior to mitosis (Liang et al., 2015). Overall, these and other observations suggest that processes upstream of pausing, such as Pol II recruitment to promoters and formation of preinitiation complexes, can be functionally decoupled from pause release. Furthermore, the function of Pol II pausing as a limiting step for transcription may be more significant not at the steady state, but during rapid responses to stimuli (Gressel et al., 2019). Observing cells during rapid responses to stimuli outside of the steady state may help reveal their regulatory architecture (Danko et al., 2013; Vihervaara et al., 2017).

A study from the Young lab showed that overexpression of c-Myc causes uniform amplification of all genes (Lin et al., 2012; Nie et al., 2012). Since c-Myc was shown to function through recruitment of P-TEFb to promoters (Rahl et al., 2010), this amplification is likely due to uniformly increased genome-wide pause release. These findings are important because they imply that the transcriptome is fundamentally modular. Throttling up of pause release siphons Pol II traffic through all available transcription start sites without altering the upstream steps such as promoter architecture. These observations reveal pausing as a central step that can globally separate regulatory inputs at distinct points of the transcription cycle. In this view, rather than integrating regulatory signals, Pol II pausing separates them.

### Regulation of the Transcriptome at the Level of Pol II Pausing

**FIGURE 3:**
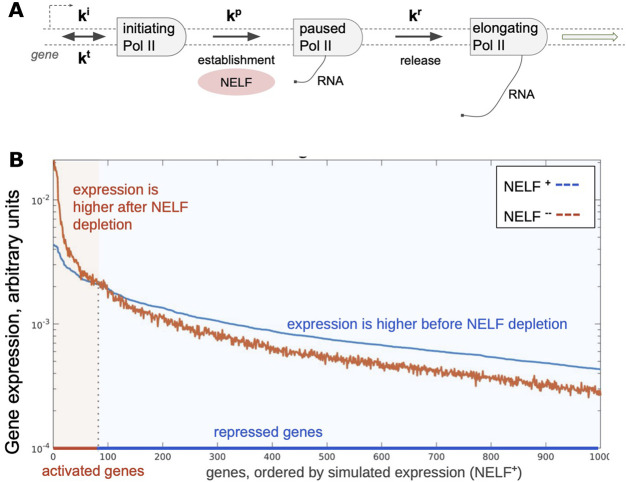
Simulated changes in gene expression upon depletion of a pausing factor. **(A)** A scheme of steps in Pol II pausing. Initiation (ki), pausing (kp), and release (kr) constants were randomly distributed for 1000 simulated genes (with cauchy and two gaussian probability distribution functions, respectively); termination constant (kt) was the same for all simulated genes. **(B)** Results of simulation showing steady-state distribution of gene expression (light colored arrow) levels with two different amounts of total available NELF in the system, which results in activation and repression of genes.

Either NELF or P-TEFb activities may become limiting at certain circumstances such as during rapid responses to stimuli. This raises a question of how these factors are redistributed across the genome when limited amounts or activity are available. This question is broadly related to an earlier concept of enhancer insufficiency (Barolo and Posakony, 2002) wherein restriction of the activator availability limits transcription from certain genes such as those with low activity promoters, thereby reducing noise. Simple simulation of a closed cell containing promoters with randomly distributed strengths shows that restricting a factor that is essential for transcription at a post-recruitment step, such as NELF, is sufficient to generate pools of genes that appear as activated and repressed (Figure 3). Addition of a second post-recruitment step such as that controlled by P-TEFb should likewise prioritize pause release among Pol II complexes that had reached the previous step. Prioritization of essential factors at multiple steps of the transcription cycle may lead to nonlinear effects including cooperativity and stabilization of transcription output. Prioritization of essential factors, we suggest, is a key principle organizing the transcriptome.

Step-wise prioritization of essential factors retrospectively accounts for known features of mammalian transcriptomes. Some transcription factors such as HSF1 and NF-kB have been shown to act on pause release by directly or indirectly recruiting P-TEFb (Zhou et al., 2012; Mahat et al., 2016; Aoi et al., 2020). P-TEFb function can be either global across the genome or involve specific groups of genes (Luo et al., 2012). Secondly, the model is agnostic to the exact promoter activity patterns and can work in all cells wherein promoter activity defines the network structure while pausing factors stabilize it. Notably, this regulation should be highly sensitive to gene copy numbers but does not require lateral interactions between gene products. This model is in principle similar to the concept of phase separation (Hnisz et al., 2017), except that the phases here are defined not spatially, but through availability of a factor that may be governed by diffusion or over-representation in different nuclear compartments. In this view, transcription of every gene must involve transitions between phases. Multiple inputs upstream and downstream of pausing including epigenetic and other broad-stroke mechanisms may form additional layers of regulation or converge on the two major limiting steps at the site of pausing. A caveat to our view is that a complex makeup of the cell is essentially reduced to a binary readout, which is certainly an oversimplification. However, the concept of binary readouts distinguishing complex systems such as cancers is gaining experimental ground (Pearson et al., 2021) and may form a paradigm for understanding and targeting these complex diseases. Transcriptional superenhancers (Hnisz et al., 2013; Hnisz et al., 2015) as well as the recently reported partitioning of small molecule drugs at distinct nuclear loci (Klein et al., 2020) must affect the specificity of globally acting factors and outcomes of their targeting through principles that remain to be explored. We note that our model is readily compatible with transcriptional bursting (Rodriguez and Larson, 2020) because bursting creates local and transient demand for transcriptional machinery components. Lastly, despite the prevalence of pause release during responses to stimuli, Pol II recruitment to promoters may still be the step limiting for overall transcription.

## Discussion

Substantial advances are made in targeting cancers based on transcription and epigenetic signatures (Malone et al., 2020). However, progress remains complicated by the inherent uncertainty in the roles of transcription machinery components and critical yet poorly understood contribution of the cellular context. Pausing regulators including NELF and P-TEFb have been proposed (Yun et al., 2018) or used as therapeutic targets including P-TEFb (CDK9) small molecule inhibitors in clinical trials (Morales and Giordano, 2016; Cassandri et al., 2020). However, the main value of pausing factors may be not in serving as therapy targets, but in providing a conceptual platform to better understand targeting of other components. Dividing the myriad factors directly or indirectly affecting transcription into those that act upstream or downstream of Pol II pausing (Figure 2) highlights the balance between these steps as an important readout of cancers that can help expose their vulnerabilities.

The process of Pol II pausing is much more granular at the molecular level than described above (Elrod et al., 2019) and includes multiple additional steps such as premature transcription termination likely involving multiple mechanisms (London et al., 1991; Brannan et al., 2012; Huang et al., 2020; Rimel et al., 2020). These and other steps should affect the balance between Pol II functions upstream and downstream of pausing. The model of transcriptome regulation by stepwise restriction of globally acting factors proposed here is agnostic to the actual nature of its components and does not need to be limited to Pol II pausing. Additional transcription elongation checkpoints such as those controlled by CDK12 (Tellier et al., 2020) may define new steps where a balance would need to be considered. Despite the ongoing quest for therapeutics targeting specific factors, cancer treatments largely rely on broad-stroke interventions and are expected to do so for the foreseeable future. Studies that integrate experiments with refined models should reveal new insights into cancer transcriptomes to improve the treatments targeting the cellular machinery *via* globally acting or general factors.
